# Neuroinflammation PET and long-term cognition and survival in symptomatic Alzheimer’s disease

**DOI:** 10.1186/s13195-025-01915-3

**Published:** 2025-11-22

**Authors:** Roos M. Rikken, Maqsood Yaqub, Emma M. Coomans, Ellen Dicks, Anne E. van der Vlies, Albert D. Windhorst, Ronald Boellaard, Yolande A.L. Pijnenburg, Everard G.B. Vijverberg, Elsmarieke van de Giessen

**Affiliations:** 1 Radiology & Nuclear Medicine, Vrije Universiteit Amsterdam, Amsterdam UMC location VUmc, Amsterdam, The Netherlands; 2 Amsterdam Neuroscience, Brain Imaging, Amsterdam, The Netherlands; 3 Alzheimer Center Amsterdam, Neurology, Vrije Universiteit Amsterdam, Amsterdam UMC location VUmc, De Boelelaan 1118, Amsterdam, 1081HV The Netherlands; 4 Amsterdam Neuroscience, Neurodegeneration, Amsterdam, The Netherlands

**Keywords:** Alzheimer’s disease, PET, Neuroinflammation, Cognition, Microglia

## Abstract

**Background:**

Neuroinflammation plays a key role in Alzheimer’s disease (AD) pathophysiology, but it is not clear how neuroinflammation contributes to disease progression. We aim to investigate the role of neuroinflammation on longitudinal cognition and survival in a unique cohort with PET imaging of translocator protein (TSPO) binding tracer [^11^C]PK11195 and long-term follow-up. We hypothesized that higher [^11^C]PK11195 binding would be associated with faster cognitive decline and higher mortality.

**Methods:**

19 participants with AD dementia, 9 participants with MCI due to AD, and 21 healthy controls (HC) with historical dynamic [^11^C]PK11195 PET data were included. Principal component analysis was performed to identify relevant [^11^C]PK11195 patterns. An additional AD ROI consisting of temporal and parietal regions was investigated. [^11^C]PK11195 scores in the principal components (PCs) and AD ROI were compared between groups using ANOVA. Longitudinal MMSE covering a period up to 11 years was used to measure cognitive decline. We used linear mixed models with random subject-specific intercepts and slopes corrected for age, sex and syndrome diagnosis to investigate the association of neuroinflammation with cognition in MCI and AD. Survival data were available for all MCI and AD participants, up to 15.7 years after PET. To examine the influence of neuroinflammation on survival time, we used age, sex, and syndrome diagnosis adjusted cox proportional-hazards models.

**Results:**

Two PCs were retained. PC1 explained 55.4% of the variance and was most explained by [^11^C]PK11195 binding in the thalamus and entorhinal cortex. PC2 explained 15.3% of the variance and constituted of mostly the entorhinal cortex. There was no difference in [^11^C]PK11195 PET between AD, MCI and HCs (range F(2) = 0.157–1.231, *P* > 0.3). [^11^C]PK11195 did not predict longitudinal MMSE (PC1: β = 0.02, *P* = 0.73; PC2: β = 0.1, *P* = 0.44; AD ROI: β = 1.3, *P* = 0.57) or survival (PC1: HR = 0.90[95%CI: 0.80, 1.03], *P* = 0.13; PC2: HR = 0.96[0.75, 1.23], *P* = 0.72; AD ROI: HR = 0.02[0.00, 1.33], *P* = 0.06).

**Conclusions:**

Contrary to our hypothesis, we did not find evidence for [^11^C]PK11195 PET predicting long-term cognitive decline or survival. This may indicate that the level of [^11^C]PK11195 PET binding earlier in the disease trajectory is not directly linked to the long-term outcome.

## Background

Neuroinflammation plays a key role in Alzheimer’s disease (AD) pathophysiology and has become a major target in drug development [1, 2]. However, the role of neuroinflammation on AD disease progression is largely unknown. It has been hypothesized that initial microglial activation triggered by amyloid-beta deposition is protective, but becomes detrimental in later stages of the disease, making it difficult to discern whether to enhance or halt microglial activity in order to achieve clinical benefit [2, 3]. Therefore, it is important to elucidate the relationship between neuroinflammation and disease progression.

Neuroinflammation and microglial density can be quantified using positron emission tomography (PET) imaging of the translocator protein (TSPO). TSPO PET studies have shown increased signal in temporal and parietal regions in AD, but associations with cognition have been inconsistent [4]. Higher TSPO PET signal has been associated with worse cognition cross-sectionally, potentially through spreading of tau [5–8]. Equally, several TSPO PET studies have found that higher TSPO PET binding was associated with steeper cognitive decline over time [9–11]. However, higher TSPO PET binding has also been associated with better clinical prognosis [12]. Moreover, microglial activation measured using CSF has been shown to protect against tau accumulation and cognitive decline [13].Besides tau, vascular pathology has been associated with higher TSPO PET signal, which may contribute to clinical progression through small vessel disease–related neuroinflammation [14, 15]. Given the inconsistent findings, it is currently not clear how neuroinflammation contributes to disease progression. In addition, most studies assessed relatively short-term follow-up, but long-term effects of neuroinflammation on cognitive decline and survival are still unknown.

Therefore, we aim to investigate the role of neuroinflammation—measured using PET with the TSPO tracer [¹¹C]PK11195—on longitudinal cognitive trajectories and survival in a unique cohort with up to 11 years of cognitive follow-up and survival data extending up to 15 years after PET. We hypothesized that [^11^C]PK11195 binding would predict longitudinal long-term cognition and survival, where higher TSPO PET binding would be associated with faster cognitive decline and higher mortality in MCI and AD dementia.

## Methods

### Participants and design

Participants with [^11^C]PK11195 PET data were included from a historical dataset [16]. Participants were only included if they signed the informed consent of the Amsterdam Dementia Cohort (ADC) (exclusion *N* = 1) [17], resulting in 9 participants with a mild cognitive impairment (MCI) due to AD, 19 with a AD dementia and 21 healthy controls (HC). In the historical dataset, diagnosis of AD dementia was based on National Institute of Neurological and Communicative Disorders and Stroke-Alzheimer’s Disease and Related Disorders Association (NINCDS-ADRDA) criteria [18], and a diagnosis of MCI on the Petersen criteria [19]. All MCI and AD participants were CSF amyloid-positive, and all HC were amyloid-negative.

### Cognition and survival data

Since no longitudinal cognitive or survival data was available from the HCs, only AD and MCI participants were included in the longitudinal cognition and survival analyses. Cognitive and survival data were extracted from the ADC and were available for all AD and MCI participants. At time of analysis, all participants had deceased (mean time PET to death: 7.8 ± 4.0 years, range: 0.4-15.7 years). Longitudinal MMSE was used to measure cognitive decline. MMSE before and after PET was used in order to more accurately estimate the slope (mean follow up (FU) time (total duration follow up with combined before and after PET): 4.35 years, median: 4.1, range: 0.1–11.7 years).

### [^11^C]PK11195 PET and MRI imaging

Detailed description of [^11^C]PK11195 PET and magnetic resonance imaging (MRI) acquisition has been described previously [16]. In brief, dynamic 60 min [^11^C]PK11195 PET images were acquired on a ECAT EXACT HR+. [^11^C]PK11195 binding potential (BP_ND_) was quantified using the basis function implementation of the simplified reference tissue model with correction for vascular binding and reference tissue time activity curves extracted using the supervised cluster analysis and were partial volume corrected using ordered subset expectation maximization algorithm as described in details in [20]. 1 T magnetic MRI were co-registered to PET images and used for ROI delineation. Next, regions of interest (ROI) were automatically defined using the Svarer atlas [21]. As neuroinflammation effects can be diffuse, we used principal component analysis (PCA) to extract relevant patterns in the form of principal components (PCs). PCA allows for the reduction of the dimensionality of the data and thereby prevent multiple testing with many ROIs. These PCs capture the main patterns in the (neuroinflammation) data, where the first components explain the largest proportion of variance in the data. Left and right regional BP_ND_ values were combined volume weighted to improve signal to noise and reduce the amount of regions and then scaled to older healthy controls (*N* = 21). The following regions from the Svarer atlas were included: entorhinal cortex, hippocampus, medial inferior temporal (lateral inferior and middle temporal gyrus), superior temporal, parietal, anterior and posterior cingulate gyrus, caudate nucleus, putamen, insula, thalamus, striatum, orbital frontal, medial inferior frontal, superior frontal, sensory motor cortex, and occipital cortex [21]. To ensure that no relevant signal was overlooked by the PCA, we included an additional AD-related ROI in a secondary analysis composed of regions previously implicated in neuroinflammation in AD (parietal cortex, hippocampus, entorhinal cortex, medial inferior temporal cortex, superior temporal cortex, posterior cingulate) [5, 6, 22].

### Statistical analysis

All statistical analyses were performed in R v.4.4.1. PCA was performed using the *princomp* package in R. PCs were retained on the Catell criterion and if the explained variance was > 10%. PC scores were compared between HCs, MCI and AD dementia groups using ANOVA. The effect of neuroinflammation on longitudinal cognition in MCI and AD dementia was examined using age, sex, and diagnosis adjusted linear mixed-effect models with subject-specific random intercepts and slopes (MMSE ~ [^11^C]PK11195*time + age + sex + diagnosis (1 + time | subject)), which were statistically considered to be the best fit based on the Akaike Information Criterion. As an exploratory analysis, linear mixed-effect models with an additional interaction between [^11^C]PK11195*time*diagnosis were run to investigate whether diagnosis had an influence on the association between [^11^C]PK11195 and longitudinal MMSE. To investigate the effect of neuroinflammation on survival, cox proportional-hazards models adjusted for age, sex, and diagnosis were performed for PC1, PC2 and the AD ROI. The non-proportionality assumption was checked for the cox models, but no non-linear terms were added to the model as it did not improve the model fit. Individuals were divided into a low and high neuroinflammation group based on a median split for visualization purposes only.

## Results

### Participant characteristics

Participant characteristics can be found in Table 1.

**Table 1:** Participant characteristics

		AD dementia (*N* = 19)	MCI (*N* = 9)	HC (*N* = 21)
Age		69 (± 7.6)	72 (± 5.5)	68 (± 7.5)
Sex, *N* female (%)		8 (42.1%)	3 (33.3%)	8 (38.1%)
MMSE		22 (± 2.6)	26 (± 1.3)	29 (± 0.73)
Hippocampal volume (mL)		6200 (± 780)	6800 (± 830)	NA
Global cortical thickness (mm)		3900 (± 270)	3900 (± 380)	NA
Fazekas score				
	0	8 (42.1%)	2 (22.2%)	0 (0%)
	1	5 (26.3%)	5 (55.6%)	0 (0%)
	2	5 (26.3%)	2 (22.2%)	0 (0%)
	Missing	1 (5.3%)	0 (0%)	21 (100%)
Microbleeds, N				
	0	16 (84.2%)	6 (66.7%)	0 (0%)
	1	2 (10.5%)	1 (11.1%)	0 (0%)
	Missing	1 (5.3%)	2 (22.2%)	21 (100%)

Left and right hippocampal volume were added. Hippocampal volume was missing in 3 AD participants (10.7%). Global cortical thickness was missing in 1 MCI and 3 AD participants (14.3%). Hippocampal volume, global cortical thickness, Fazekas score and microbleeds score were not available for the HCs

### [^11^ C]PK11195 PET-derived principal components

**Fig. 1:**
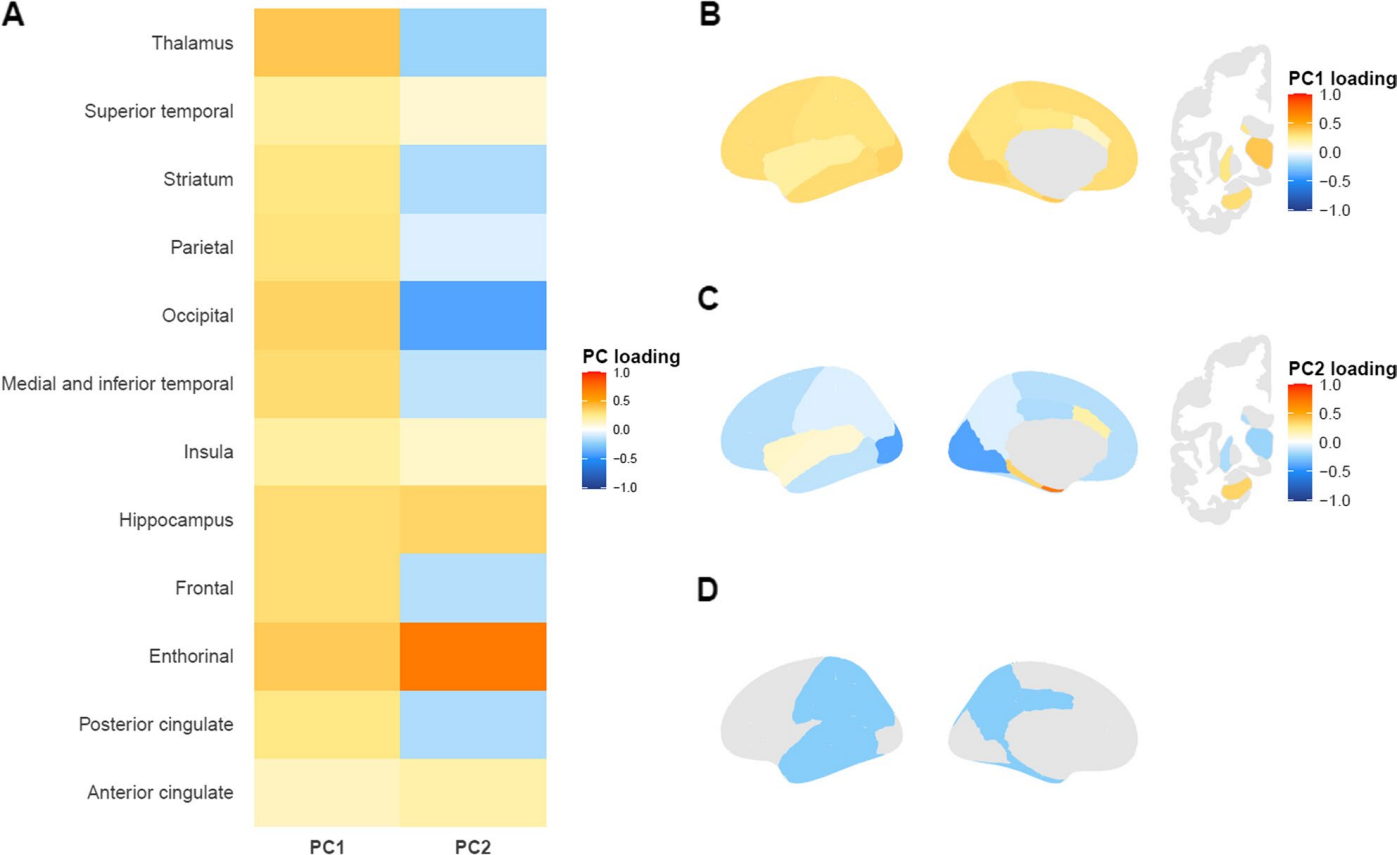
Visualization of [^11^C]PK11195 PET-derived principal components and AD ROI. **A.** Loading of PC1 and PC2 onto all included brain regions. **B. **Loading of PC1 onto all included brain regions. **C. **Loading of PC2 onto all included brain regions. **D. **Regions included in the AD ROI. The ggseg package was used to visualize the loading of the PCs and the regions included in the AD ROI. *Abbreviations*: PC = principal component, ROI = region of interest

**Fig. 2:**
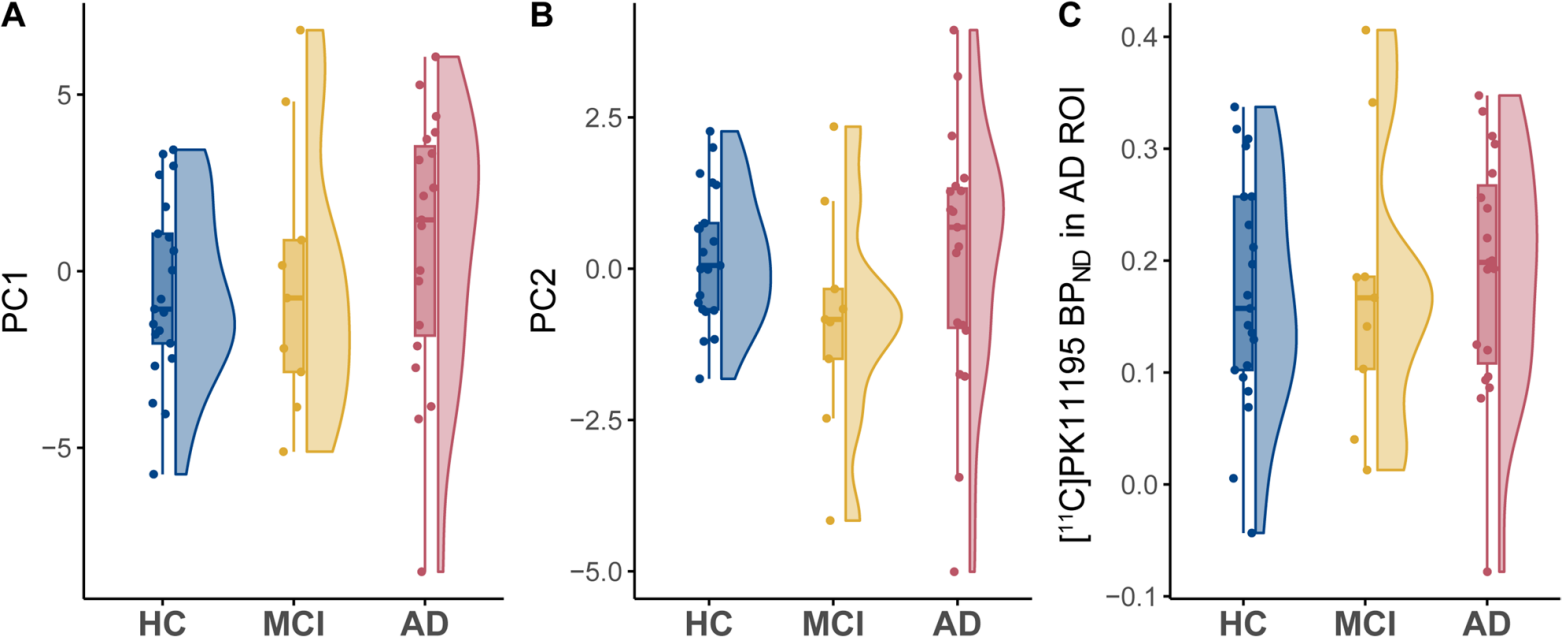
Comparison of [^11^C]PK11195 PET between HC, MCI and AD dementia. Group comparisons were assessed by one-way ANOVA. No significant differences were found. PC1 and PC2 reflect component scores. *Abbreviations*: PC = principal component, ROI = region of interest, HC = healthy control, MCI= mild cognitive impairment, AD = Alzheimer’s disease

PC1 and PC2 explained 55.4% and 15.3% of the variance respectively (Fig. 1**)**. PC1 mainly consisted of the thalamus and entorhinal cortex. PC2 represented opposing signal in the entorhinal cortex and hippocampus versus the thalamus and occipital cortex. There was no significant difference in the [^11^C]PK11195 PET derived PCs between the HC, MCI and AD dementia group (PC1: F(2) = 0.78, *P* = 0.46; PC2: F(2) = 1.23, *P* = 0.30) (Fig. 2AB). There was also no difference in [^11^C]PK11195 BP_ND_ AD ROI between HC, MCI and AD dementia group (F(2) = 0.157, *P* = 0.86) (Fig. 2C).

### [^11^C]PK11195 PET and longitudinal cognition

**Fig. 3:**
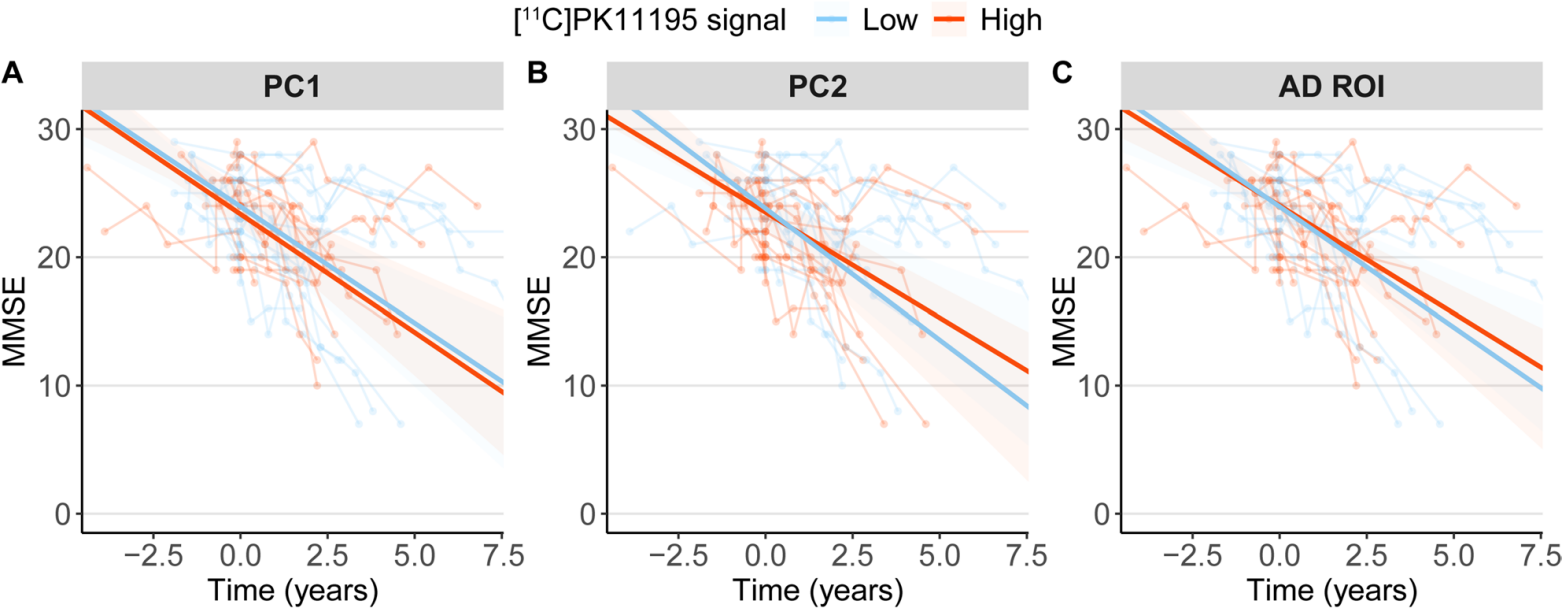
[^11^C]PK11195 PET and longitudinal MMSE in A. PC1, B. PC2 and C. AD ROI. Neuroinflammation PET was dichotomized for each panel into low and high based on median split for plotting only. *Abbreviations*: PC = principal component, MMSE = Mini Mental State Examination, ROI = region of interest, MCI = mild cognitive impairment, AD = Alzheimer’s disease

There was no significant association between [^11^C]PK11195 PET in PC1 or PC2 or in the combined model with PC1 and PC2 together on longitudinal MMSE in MCI and AD combined (model without HC) (PC1: β = 0.02, *P* = 0.79; PC2: β = 0.1, *P* = 0.45; PC1 + PC2: β (PC1) = 0.09, *P* = 0.48, β (PC2) = 0.01, *P* = 0.88) (Fig. 3AB), while the group showed significant decline on MMSE over time (PC1 model: β (time)= −1.8, *P* < 0.001; PC2 model: β (time)= −1.8, *P* < 0.001). There was a significant effect of diagnosis (PC1 model diagnosis: β = 4.6, *P* < 0.001; PC2 model diagnosis: β = 5.1, *P* < 0.001). Therefore, we also performed a linear mixed model with an extra interaction term (PC*time*diagnosis) as an exploratory analysis. There was no significant interaction effect with diagnosis of any PC on longitudinal MMSE (PC1: β= −0.05, *P* = 0.60; PC2: β= −0.07, *P* = 0.70), indicating that the association between the principal components and MMSE over time did not differ significantly between groups. Similarly, [^11^C]PK11195 PET in the AD ROI also was not significantly associated with longitudinal MMSE (β = 1.15, *P* = 0.62) (Fig. 3C). Again, there was a significant effect of diagnosis (β = 4.7, *P* < 0.001), but no significant interaction effect of [^11^C]PK11195*time with diagnosis (β= −1.3, *P* = 0.70).

### [^11^C]PK11195 PET and survival

**Fig. 4:**
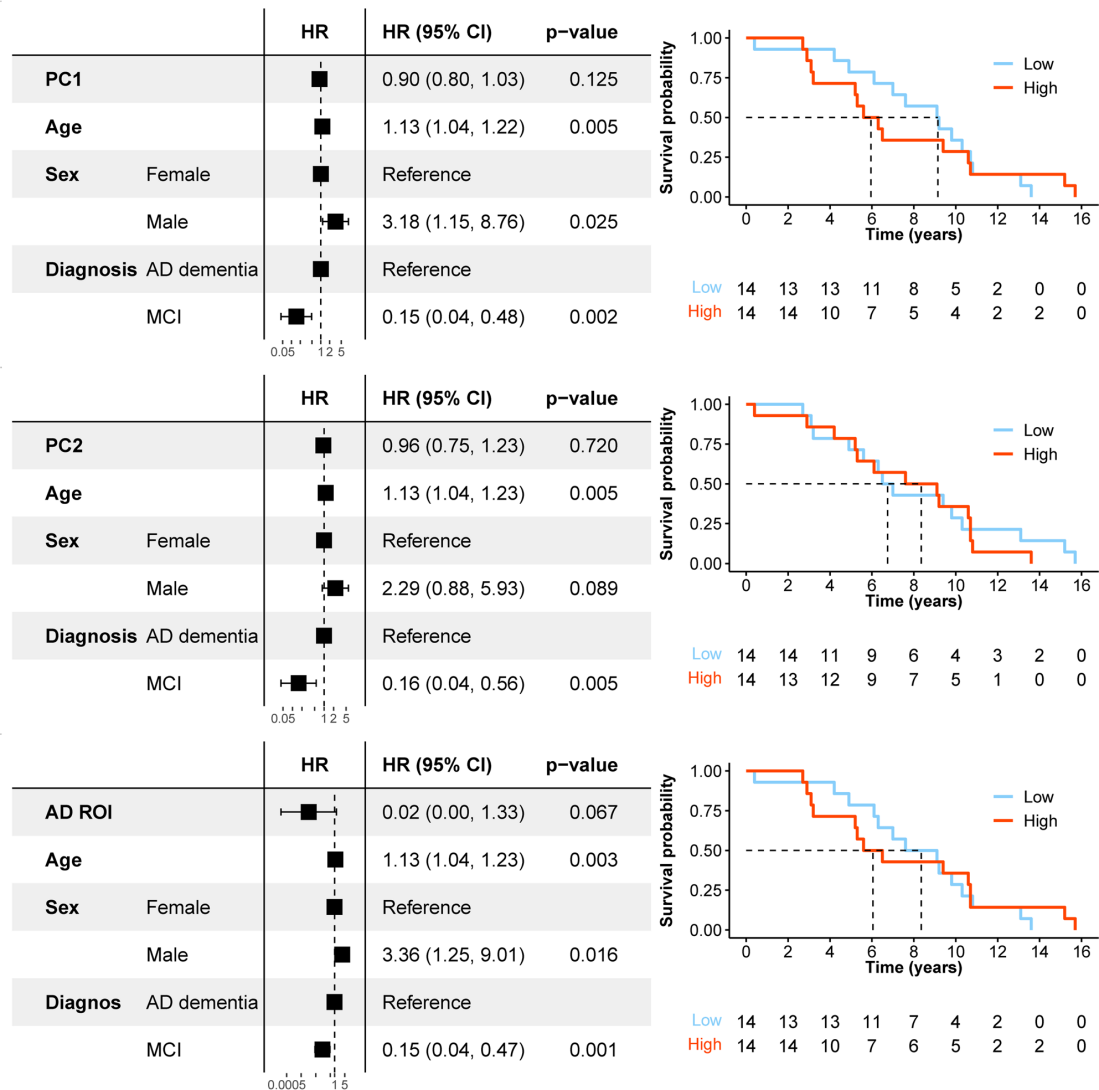
Cox proportional-hazards models to evaluate the influence of [^11^C]PK11195 PET on mortality. Neuroinflammation PET was dichotomized for each panel into low and high based on median split for plotting only. *Abbreviations*: PC = principal component, HR = Hazard ratio, CI = Confidence interval, MMSE = Mini Mental State Examination, ROI = region of interest, MCI = mild cognitive impairment, AD = Alzheimer’s disease

In MCI and AD combined, the median survival time was 7.3 years. Cox models showed no significant effect of PC1 (HR = 0.90 [95%CI: 0.80, 1.03], *P* = 0.13) or PC2 (HR = 0.96[0.75, 1.23], *P* = 0.72) on survival (Fig. 4). In both models, higher age was associated with increased mortality risk (PC1 model age: 1.13[1.04, 1.22], *P* = 0.005; PC2 model age: 1.13[1.04, 1.23], *P* = 0.005) and an MCI diagnosis with decreased mortality risk (PC1 model diagnosis: 0.15[0.04, 0.48], *P* = 0.002; PC2 model diagnosis: 0.16 [0.04, 0.56], *P* = 0.005). The combined model (PC1 + PC2) (PC1: HR = 0.91 [95%CI: 0.79, 1.03], *P* = 0.14; PC2: (HR = 0.99[0.78, 1.26], *P* = 0.95) or [^11^C]PK11195 PET in the AD ROI (HR = 0.02[0.00, 1.33], *P* = 0.06) also showed no significant associations with survival (Fig. 4).

## Discussion

In contrast to our hypothesis, we did not find a predictive effect of neuroinflammation measured by [^11^C]PK11195 PET on long-term cognition or survival in AD. In the present study we identified two PCs derived from [^11^C]PK11195 that explained most of the variance in the data. PC1 mainly consisted of the thalamus and entorhinal cortex. PC2 represented opposing signal in the entorhinal cortex and hippocampus versus the thalamus and occipital cortex. [^11^C]PK11195 retention in both the PCs and the AD ROI did not associate with decline in MMSE or mortality over a maximum of 11.7 years of MMSE data and a maximum of 15.7 years of follow-up for mortality.

In line with the previously reported results on the same data [16], we did not find differences in [^11^C]PK11195 PET between MCI, AD dementia and HCs. In a meta-analysis, increased TSPO PET signal was found throughout the brain in AD and less evident in MCI [4]. Similarly, some studies reported increased TSPO in AD dementia stage, but not in the MCI stage [23]. However, other studies have reported higher TSPO PET signal specifically in prodromal stages [24]. The fact that we did not find differences in [^11^C]PK11195 PET between MCI, AD dementia and HCs may be partly attributed to the low signal to noise ratio of [^11^C]PK11195, which may have contributed to higher variability in the signal in the HC group. Nevertheless, some studies using second generation TSPO tracers with generally better signal to noise ratios ([^18^F]DPA-714, [^11^C]PBR28, [^18^F]FEDAA1106 & [^18^F]FEPPA) could also not find significant differences in TSPO PET signal between AD and controls [25–28], while others found higher TSPO PET binding in AD compared to controls [4, 5, 22, 24]. These studies illustrate the discrepancy in TSPO PET findings in AD, which may be explained by differences in methodology, and highlights the need for replication.

TSPO PET predicted longitudinal cognition in previous studies [9–11]. In a sample of stable cognitively unimpaired (CU), progressing CU and AD, higher whole cortical [^11^C]PK11195 standardized uptake value ratio (SUVR) was found to predict steeper decline on Knight Alzheimer’s Disease Research Center preclinical AD cognitive composite (ADRC-PACC) score [10]. Similarly, in a sample consisting of CU, MCI and AD dementia, higher anterior temporal [^11^C]PK11195 PET signal predicted steeper cognitive decline measured by Addenbrooke’s Cognitive Examination-revised (ACE-R) [9]. In a study using a second-generation TSPO tracer, [^11^C]DPA-713 BP_ND_ was found to be the best predictor of decline in global cognition and memory in MCI and AD dementia patients, where again higher PET signal was related to steeper cognitive decline [11]. However, the opposite association has also been reported that higher TSPO binding was associated with slower decline on MMSE in prodromal AD and AD [12]. The discrepancy in findings may be explained by multiple factors. Again, there are differences in TSPO tracer used which have different signal to noise ratios and also use different quantification methodologies. Moreover, two of the aforementioned studies included CU participants in their analyses, which we were not able to do because this data was not available. In addition, the present study investigated longitudinal cognition with a longer follow up period compared to previous studies, which provides an accurate picture of long-term decline but might make it more difficult to predict cognition based on TSPO PET levels acquired many years prior. Finally, in our study MMSE was used as an outcome measure, which was not always used as a main cognitive outcome in the aforementioned studies. Replication studies or long-term follow-up of the previous studies are necessary to elucidate the associations between neuroinflammation PET and longitudinal cognition. Moreover, future studies should include participants throughout the AD continuum and other measures of amyloid and tau pathology to hopefully shed light on the associations between neuroinflammation and cognition at different disease stages.

In addition to cognitive outcomes, associations of TSPO PET with clinical severity have shown conflicting results [12, 23, 29, 30]. Higher increases in TSPO PET binding were related to cognitive worsening on CDR-SB and longitudinal increases in TSPO binding was found to be higher in patients with clinical progression compared to those without progression [29]. On the other hand, another study found higher TSPO PET binding to be associated with better clinical prognosis [12]. In the present study we could not find significant associations between [^11^C]PK11195 signal and mortality. This could potentially be explained by the differential associations of TSPO PET and clinical progression in different disease stages, where higher initial TSPO PET signal is thought to be protective, but later increases in TSPO PET signal are thought to be detrimental [2, 3, 12, 24, 31].

A major strength of the present study is the unique dataset with long cognitive and clinical follow up spanning almost 16 years. Additionally, since we used historical data, mortality data was available in all participants. This study also has several limitations. First, the sample size of the total group that underwent historic [^11^C]PK11195 PET is relatively small and therefore the results should be interpreted with caution. Nevertheless, studies with similar or smaller sample sizes were able to detect effects [10, 11, 29]. Moreover, there was extensive longitudinal cognitive follow up, allowing for a relatively large amount of data for the amount of individuals included and a more accurate depiction of cognitive decline. Second, we used historic [^11^C]PK11195 data. While there is no genotype effect present, [^11^C]PK11195 has a low signal to noise ratio. Hence, we may have less sensitivity to detect more subtle differences. Third, we used MMSE as our cognitive outcome due to the large amount of missing data in further neuropsychological testing. The MMSE may not be the most sensitive measure to capture cognitive and functional decline in AD compared to a composite including more domains [32, 33]. Fourth, TSPO PET may not entirely capture the neuroinflammatory processes involved in AD. TSPO PET was suggested to represent microglial density, rather than activation, and was found to also be present on endothelial cells and astrocytes, although the binding is largely explained by binding to microglia [34–36]. These factors and the additional low signal to noise ratio complicate the interpretation of [^11^C]PK11195 PET as a measure of microglial activation. In addition, TSPO PET cannot differentiate between microglial states, which may be important for disentangling the complex relation between neuroinflammation and cognition. Finally, we cannot rule out that co-pathologies, particularly vascular changes, may have influenced longitudinal cognition and survival, and that the interplay between co-pathologies and neuroinflammation could have affected the predictive value of baseline [^11^C]PK11195 PET over the extended follow-up period.

## Conclusions

In conclusion, contrary to our hypothesis, [^11^C]PK11195 PET did not predict longitudinal cognition or survival. This may indicate that [^11^C]PK11195 PET earlier in the disease trajectory is not directly linked to the long-term outcome. Future research with larger sample sizes, longitudinal neuroinflammation PET, and focus on tracers with higher signal to noise ratio and tracers that are more specific to anti- or pro-inflammatory peaks in AD is necessary to further establish this finding and help shedding light on the complex relationships between neuroinflammation and longitudinal cognition and clinical progression.

## Data Availability

Data can be made available upon reasonable request.

## Abbreviations

AD: Alzheimer’s disease
ADC: Amsterdam Dementia Cohort
CU: Cognitively unimpaired
FU: Follow-up
HC: Healthy control
HR: Hazard ratio
MCI: Mild Cognitive Impairment
MMSE: Mini mental state examination
MRI: Magnetic Resonance Imaging
PCA: Principal component analysis
PC: Principal component
PET: Positron Emission Tomography
ROI: Region of interest
TSPO: Translocator protein
